# Intracellular *Theileria* Parasites PIN Down Host Metabolism

**DOI:** 10.3389/fcell.2020.00134

**Published:** 2020-03-17

**Authors:** Souhila Medjkane, Jonathan B. Weitzman

**Affiliations:** Université de Paris, UMR 7216 Epigenetics and Cell Fate, CNRS, Paris, France

**Keywords:** apicomplexa, parasite, Pin1, *Theileria*, PKM2, Fbw7, Warburg, metabolism

## Introduction

The word “*parasite*” has been used since the 16th century, stemming from the Greek *parasitos* meaning “*eating at another's table,”* from para- meaning “*alongside”* + sitos “*food.”* The relationship between parasites and their hosts is an inherently metabolic one. Intracellular parasites feed off their host and are therefore likely to perturb their metabolic pathways in the process. Indeed, there has been much recent interest in the role of metabolic exchange in host-parasite interactions (Blume and Seeber, 2018; Zuzarte-Luís and Mota, 2018; Krishnan et al., 2019). In recent years we have become fascinated by a remarkable metabolic host-parasite interaction; *Theileria* parasites can reprogram their host cells to drive a cancer-like metabolic state. And our most exciting discovery has been the critical role that the Peptidyl-Prolyl *Cis/Trans* Isomerase Pin1 plays in this relationship.

## Talented *Theileria* Parasites

*Theileria* spp. are obligate, intracellular parasites belonging to the phylum of apicomplexa. Two *Theileria* species, *T. parva*, and *T. annulata* are bovine-specific pathogens that cause disease with considerable economic impact due to the high cost of treatment, the cost of anti-tick control, animal mortality, and decreased bovine production. Tropical Theileriosis kills over 1.1 million cattle per year and costs in the hundreds of millions of dollars. Infection by *Theileria* causes a lymphoproliferative disease in cows that has some clinical features of human leukemias (Tretina et al., 2015). *T. annulata* infects bovine B cells and macrophages, whereas the related species *T. parva* infects B and T lymphocytes. *Theileria*-infected cells are transformed and immortalized (Cheeseman and Weitzman, 2015; Tretina et al., 2015); they display cancer phenotypes such as uncontrolled proliferation, growth factor independence, and increased invasiveness and the ability to form metastases in immunodeficient mice (Tretina et al., 2015). Of particular interest, *Theileria*-dependent transformation is reversible; animals can be cured by treatment with the theilericidal drug Buparvaquone. Incubating *Theileria*-infected cells *in vitro* with Buparvaquone, diminishes the number of intracellular parasites in host leukocytes, which loose the transformed phenotypes, stop proliferating, and regain apoptosis sensitivity. To drive host cell transformation, the parasite manipulates the host cell signaling pathways that control cell proliferation and survival. Several signaling pathways were implicated, including c-Jun N-terminal Kinase (JNK) and host nuclear factors c-Myc, NF-κB, and AP-1 (Chaussepied et al., 1998; Heussler et al., 2002; Dessauge et al., 2005; Tretina et al., 2015). We showed that the Jun/AP-1 transcription factor maintains a critical oncogenic microRNA feedback loop (Marsolier et al., 2013). Another fascinating feature of *Theileria*-induced transformation is the induction of a metabolic signature characteristic of the “Warburg effect” observed in cancer cells (hereafter referred to as a Warburg-like effect) (Cairns et al., 2011; Medjkane and Weitzman, 2013; Medjkane et al., 2014; Metheni et al., 2015). The parasite-induced Warburg-like effect shows the classic signs of a shift from oxidative phosphorylation to aerobic glycolysis. We and others previously reported the central role of the Hypoxia-inducible factor 1 alpha (HIF1α) in driving the expression of glycolytic enzymes and metabolic genes in infected cells (Medjkane et al., 2014; Metheni et al., 2015). Despite this progress in identifying host pathways underlying the transformed phenotype, it remained unclear how the intracellular parasite initiates the signaling events leadings to rewiring of the host transcriptome.

## UnderPINning Host-Parasite Interaction

In order to identify potential secreted oncoproteins in the *T. annulata*, we mined the parasite genome looking for genes encoding proteins with signal peptides that might be secreted into the host cytoplasm and acts as “epigenators” (Cheeseman and Weitzman, 2015) of oncogenic signals to hijack host regulatory pathways. A bioinformatics pipeline led to a relatively restricted list of candidate genes and the most promising on the list was the parasite homolog of Phosphorylation-Dependent Peptidyl-Prolyl *Cis/Trans* Isomerase PIN1 (Marsolier et al., 2015). The role of human Pin1 in carcinogenesis and metabolic reprogramming offered a link between infection and transformation by *Theileria* parasites (Nakatsu et al., 2019). The parasite encoded isomerase, that we named TaPin1, is particularly interesting; it has a catalytic isomerase domain and the WW domain present in mammalian Pin1 is replaced by a putative signal peptide sequence. While several Pin1 homologs also lack the WW domain, the PPIase domain of TaPin1 is well conserved. Indeed, the TaPin1 PPIase domain shares 47% identity with hPin1, 45% with *Arabidopsis thaliana* AtPin1, and 43% with *Trypanosoma brucei* TbPin1 (Marsolier et al., 2015). Interestingly, the signal peptide is not conserved in non-transforming species of *Theileria* or in the related apicomplexan homologs in *Toxoplasma* or *Plasmodium* (Marsolier et al., 2015). We showed that the TaPin1 protein is a *bona fide* prolyl isomerase and that it is secreted into host cells (Marsolier et al., 2015). The importance of TaPin1 in the parasite-induced transformation process was highlighted by the discovery that TaPin1 isomerase activity can be inhibited by the anti-parasite drug Buparvaquone. An additional twist was the finding that Buparvaquone-resistant parasites have a mutation in the gene encoding TaPin1. The same A53P mutation has now been reported in drug-resistant isolates from both Tunisia and Sudan (Marsolier et al., 2015; Salim et al., 2019). This mutation affects the ability of Buparvaquone to enter into the active site and inhibit isomerase activity. Interestingly, the presence of a signal peptide was observed only in the transforming species (*T. annulata* and *T. parva*), but not in non-transforming species or closely related apicomplexan such as *Plasmodium* or *Toxoplasma* (Marsolier et al., 2015). Although there are likely to be other parasite-encoded proteins that contribute to the transformation of the host cells, TaPin1 represents a remarkable of example of how a prolyl isomerase has evolved to play a key role in host-parasite relationships.

## TaPin1, a Molecular Lynchpin

**Figure 1:**
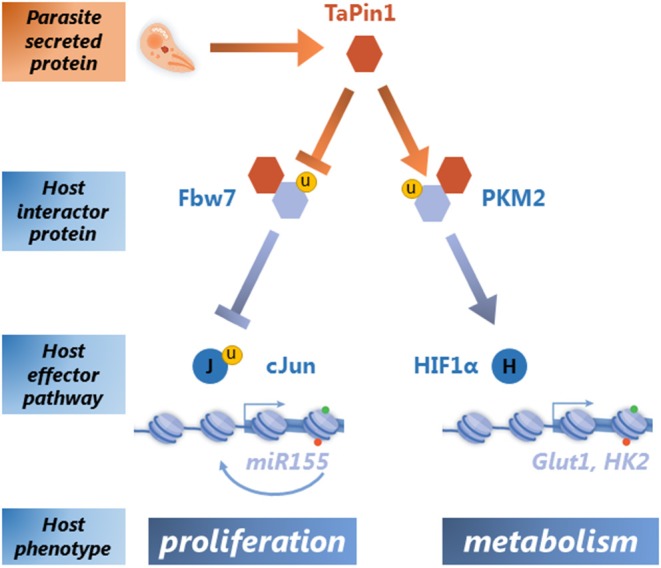
Secreted TaPin1 isomerase regulates host signaling pathways leading to proliferative and metabolic phenotypes. A schematic representation of the role of the secreted TaPin1 molecule in host-parasite interactions. *Theileria* parasites (represented in orange) secrete TaPin1 proteins into the host cell (represented in blue). The TaPin1 interacts with two host signaling pathways: by destabilizing the Fbw7 ubiquitin ligase, TaPin1 activates the c-Jun transcription factor leading to regulation of proliferative genes such as the onco-miR-155 (Marsolier et al., 2013, 2015); in contrast, TaPin1 stabilizes the host PKM2 protein which drives host cell metabolic genes through the transcription factor HIF1α (Medjkane et al., 2014; Marsolier et al., 2019). The yellow circle indicates the importance of ubiquitination in the TaPin1-regulated pathways.

Once TaPin1 was identified as a critical parasite-secreted epigenator, the question remained how it could hijack host cell signaling pathways. Pin1 is a conserved enzyme that specifically isomerizes phosphorylated Ser/Thr-Pro bonds in a defined subset of proteins, thereby inducing conformational changes impacting their stability, localization and activity. Human Pin1 protein has multiple substrates involved in a wide range of cellular processes that contribute to transformation (Marsolier and Weitzman, 2014; Zhou and Lu, 2016). A search for TaPin1 interactors and host partner proteins identified at least two host pathways that are induced by the parasite isomerase (Figure 1). We showed that the TaPin1 protein interacts with host ubiquitin ligase Fbw7, leading to its auto-degradation (Marsolier et al., 2015). This interaction releases the host oncoprotein c-Jun from Fbw7-dependent ubiquitination and degradation. The c-Jun protein is part of the AP-1 transcription factor that induces the oncomiR-155 which drives host cell proliferation (Marsolier et al., 2013). AP-1 also induces the gene encoding the matrix metalloprotease MMP-9 which drives host cell invasive phenotypes (Cock-Rada et al., 2012). We also identified the host protein Pyruvate Kinase isoform M2 (PKM2), which is critical for the Warburg-like effect and the transcription of glycolytic enzymes in cancer cells, as a TaPin1 interactor (Marsolier et al., 2019). This time the consequence is the stabilization of PKM2 which leads to HIF-1α-dependent regulation of host metabolism. The TaPin1-PKM2-HIF-1α axis causes induction of host metabolic enzymes (such as GLUT1 and Hexokinase 2), increased glucose uptake and the transformed phenotypes of parasite-infected cells (Medjkane et al., 2014; Marsolier et al., 2019). These are the combined features of the parasite-induced Warburg-like effect. The precise molecular mechanisms by which TaPin1 stabilizes host PKM2 protein, while promoting Fbw7 degradation, is unclear. We hypothesize that the prolyl isomerisation of PKM2 or Fbw7 could differentially affect the interaction with ubiquitin ligases or other factors that modulate protein stability.

## Discussion

Many studies on the role of the Pin1 phosphorylation-dependent Peptidyl-Prolyl *Cis/Trans* Isomerase have firmly placed the protein as a key regulator of oncogenic and metabolic pathways (Marsolier and Weitzman, 2014; Zhou and Lu, 2016; Nakatsu et al., 2019). The discovery and characterization of the parasite TaPin1 add parasite-host interactions to the list of effects of this multi-tasking enzyme. As described above, TaPin1 links parasitism to the regulation of host metabolism and host cell proliferation. Our findings on TaPin1 binding and isomerization of host substrates converge on the regulation of strategic host transcriptional reprogramming leading to two major biological processes that offer clear advantages for the parasite (Figure 1). First, TaPin1 contributes to host cell proliferation and tumor growth via stabilization of c-Jun which promotes transformation, thereby enabling parasite dissemination. Secondly, TaPin1 induces major metabolic reprogramming through activation of the PKM2-HIF1α axis. This shift in cellular glucose resources could potentially provide critical nutrients required for *Theileria* proliferation and maintenance within the host cells. Interestingly, the acquisition during evolution of a signal peptide for TaPin1 that is restricted to transforming *Theileria* species (*T. annulata* and *T. parva*) provides a compelling way to be secreted into the cytoplasmic host compartment in order to hijack transduction pathways and rewire host transcriptional programs. In this way TaPin1 is critical for parasite survival and is a promising drug target. Indeed, the observation in the field of Buparvaquone-resistant parasites and mutations in the *TaPin1* gene highlights the need for alternative Pin1 inhibitors that can still target mutant proteins. The levels of host bovine *BtPin1* transcripts and protein were unaffected by Buparvaquone treatment, suggesting that this drug specifically targets the parasite protein and this might explain the absence of toxicity in uninfected cells. Of note, Juglone, a well-characterized inhibitor of mammalian Pin1 can substitute for the treatment by Buparvaquone leading to a decrease in parasite burden and viability of host cells infected with *T. annulata* or *T. parva in vitro* (Marsolier et al., 2015). Clearly, Pin1 proteins from different species will continue to amaze us with their versatility and multi-tasking in the years ahead. This is likely to remain an exciting field, with clinical relevance for both cancer and infectious diseases.

## Author Contributions

SM and JW wrote the article.

### Conflict of Interest

The authors declare that the research was conducted in the absence of any commercial or financial relationships that could be construed as a potential conflict of interest.
